# Structural and Kinetic Studies of the Human Nudix Hydrolase MTH1 Reveal the Mechanism for Its Broad Substrate Specificity*[Fn FN2]

**DOI:** 10.1074/jbc.M116.749713

**Published:** 2016-12-29

**Authors:** Shaimaa Waz, Teruya Nakamura, Keisuke Hirata, Yukari Koga-Ogawa, Mami Chirifu, Takao Arimori, Taro Tamada, Shinji Ikemizu, Yusaku Nakabeppu, Yuriko Yamagata

**Affiliations:** From the ‡ Graduate School of Pharmaceutical Sciences, Kumamoto University, Kumamoto 862-0973,; the § Priority Organization for Innovation and Excellence, Kumamoto University, Kumamoto 862-0973,; the ¶ Quantum Beam Science Research Directorate, National Institutes for Quantum and Radiological Science and Technology, Tokai, Ibaraki 319-1106, and; the ‖ Medical Institute of Bioregulation, Kyushu University, Fukuoka 812-8582, Japan

**Keywords:** 8-oxoguanine (8-oxoG), crystallography, enzyme catalysis, enzyme kinetics, enzyme mechanism, enzyme mutation, substrate specificity

## Abstract

The human MutT homolog 1 (hMTH1, human NUDT1) hydrolyzes oxidatively damaged nucleoside triphosphates and is the main enzyme responsible for nucleotide sanitization. hMTH1 recently has received attention as an anticancer target because hMTH1 blockade leads to accumulation of oxidized nucleotides in the cell, resulting in mutations and death of cancer cells. Unlike *Escherichia coli* MutT, which shows high substrate specificity for 8-oxoguanine nucleotides, hMTH1 has broad substrate specificity for oxidized nucleotides, including 8-oxo-dGTP and 2-oxo-dATP. However, the reason for this broad substrate specificity remains unclear. Here, we determined crystal structures of hMTH1 in complex with 8-oxo-dGTP or 2-oxo-dATP at neutral pH. These structures based on high quality data showed that the base moieties of two substrates are located on the similar but not the same position in the substrate binding pocket and adopt a different hydrogen-bonding pattern, and both triphosphate moieties bind to the hMTH1 Nudix motif (*i.e.* the hydrolase motif) similarly and align for the hydrolysis reaction. We also performed kinetic assays on the substrate-binding Asp-120 mutants (D120N and D120A), and determined their crystal structures in complex with the substrates. Analyses of bond lengths with high-resolution X-ray data and the relationship between the structure and enzymatic activity revealed that hMTH1 recognizes the different oxidized nucleotides via an exchange of the protonation state at two neighboring aspartate residues (Asp-119 and Asp-120) in its substrate binding pocket. To our knowledge, this mechanism of broad substrate recognition by enzymes has not been reported previously and may have relevance for anticancer drug development strategies targeting hMTH1.

## Introduction

DNA is exposed to oxidative stress either directly or via oxidation of its precursor dNTP in the nucleotide pool. Among the various types of oxidized dNTPs, 8-oxo-dGTP is the major oxidized mutagenic nucleotide because it is efficiently inserted opposite adenine as well as cytosine on the template by DNA polymerases (1). Thus, its accumulation during aging is believed to be a major cause of cancer (2, 3). For repair of the oxidative damage in DNA, there are a number of excision repair mechanisms that remove the damaged bases or nucleotides. In the nucleotide pool, which is more susceptible to oxidation by reactive oxygen species than DNA (4, 5), human MutT homolog 1 (hMTH1, human NUDT1) is the most prominent mammalian enzyme among other enzymes responsible for sanitization of the nucleotide pool (6–10).

hMTH1 is localized both in the nucleus and in mitochondria (11) where it protects cells from spontaneous mutagenesis, carcinogenesis, and degenerative disorders such as neurodegeneration that are induced by oxidized DNA precursors (12–15). Recently, hMTH1 inhibitors were proposed as anticancer therapeutics (16, 17) because the expression of hMTH1 is up-regulated in a wide variety of tumors (18–21) that show a high level of oxidative stress. Therefore, hMTH1 is crucial for survival of cancer cells (to avoid incorporation of oxidized nucleotides that induce DNA damage and cell death) but is considered nonessential in normal cells (16, 17). Nevertheless, the role of hMTH1 in cancer cells and usefulness of hMTH1 inhibition remain unclear (22).

hMTH1 is characterized by broad substrate specificity as compared with *Escherichia coli* MutT; that is, hMTH1 hydrolyzes different oxidized nucleotides such as 8-oxo-dGTP, 2-oxo-dATP, and 8-oxo-dATP with almost the same efficiency (23, 24), whereas MutT shows high substrate specificity for 8-oxoguanine nucleotides. 2-Oxo-dATP is also known to be a mutagenic substrate just as 8-oxo-dGTP because the misincorporation of 2-oxo-dATP into DNA induces G:C to T:A transversion mutations (25). Accordingly, hMTH1 prevents mutations and cell dysfunction by its hydrolytic activity toward both 2-oxo-dATP and 8-oxo-dGTP. This is the reason why MutT cannot completely reverse the cytotoxicity of either TH287 or TH588, the new hMTH1 inhibitors designed as anticancer drugs (16). Thus, the mechanisms of substrate recognition by and binding of inhibitors to hMTH1 are of biological interest and important for effective drug development.

There remain some questions regarding how hMTH1 can discriminate oxidized bases from normal bases, and moreover, can recognize different types of oxidized nucleotides in the same substrate binding pocket. Although the previous structural and mutational studies on hMTH1 have revealed its scheme of recognition of 8-oxo-dGTP and the significant contribution of the neighboring aspartate residues (Asp-119 and Asp-120) (26–30), a detailed mechanism underlying the broad substrate specificity of hMTH1 remains unclear because the crystal structure of hMTH1 complexed with 2-oxo-dATP (being a good substrate) is unknown and because almost all the known structures have been obtained at low pH (<4.5) (16, 17, 22, 29–31), and it would be difficult to discuss the protonation state of the key aspartate residues (Asp-119 and Asp-120) under such acidic conditions.

Here, we report the crystal structures of hMTH1 at neutral pH in complex with the major substrates, 8-oxo-dGTP and 2-oxo-dATP, at 1.21- and 1.20-Å resolution, respectively, using our hMTH1(G2K) mutant (it contains a homogeneous N terminus), which produces a new crystal form while retaining the hydrolytic activity (32). These crystal structures showed clear electron densities of the ligands, including the triphosphate moiety that bind to the Nudix motif (hydrolase motif) and align for the hydrolysis reaction. Furthermore, the protonation state of the neighboring aspartate residues (Asp-119 and Asp-120) in the substrate binding pocket was found to be different for the recognition of 8-oxo-dGTP and 2-oxo-dATP, according to the bond length analysis of the aspartate residues using high-resolution X-ray data, and also the kinetic and structural analysis of the Asp-120 mutants. Finally, we illustrated how this unique mechanism results in the broad substrate specificity of hMTH1.

## Results and Discussion

### 

#### 

##### Overall Structures of Binary Complexes of hMTH1 with Oxidized Purine Nucleotides

To understand the catalytic mechanism of hMTH1 including the protonation state of the Asp residues (Asp-119 and Asp-120) in the substrate recognition mode and the binding mode of the triphosphate moiety in the Nudix motif with the Glu cluster, we determined the crystal structures of binary complexes of hMTH1(G2K) with 8-oxo-dGTP or 2-oxo-dATP at neutral pH. We previously reported that hMTH1(G2K) (it has a homogeneous N terminus) shows a new crystal form with high diffraction quality (1.2 Å) at neutral pH. In addition, the catalytic activity of hMTH1(G2K) toward 8-oxo-dGTP is almost identical to that of the wild type (32). Hereafter, hMTH1(G2K) is referred to as hMTH1 or the wild type for simplicity.

**FIGURE 1.**
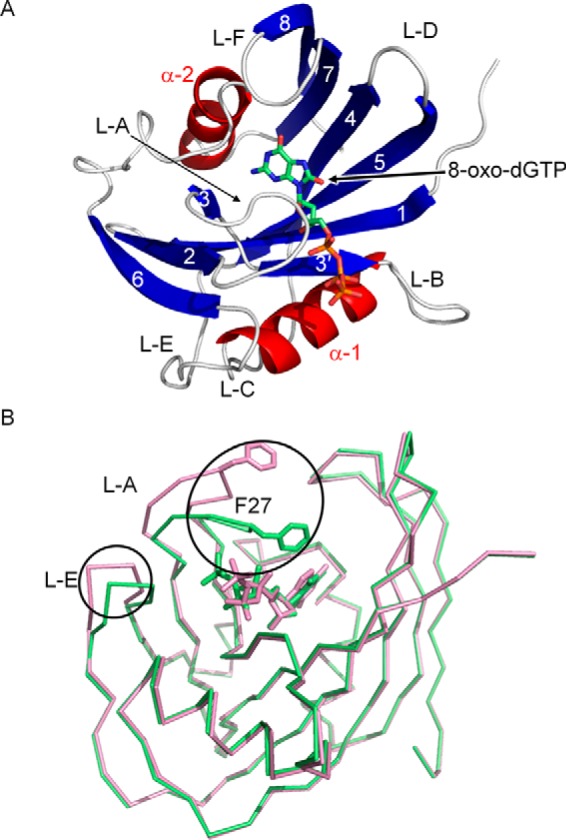
**Structure of hMTH1.** *A,* overall structure of hMTH1 is shown as a schematic representation (α-helices are shown in *red*, β-strands in *blue*), and 8-oxo-dGTP is shown as a *stick model*. Secondary structure elements (α-1, α-2, and β-1 through β-8) and loop regions (L-A through L-F) are labeled. *B,* comparison of the structures of the complex with 8-oxo-dGTP (*green*) and complex with 2-oxo-dATP (*pink*). L-A and L-E, which show different conformations between the complexes, are *circled*.

In the crystals of the hMTH1 complexes, there are two molecules per asymmetric unit. The overall structures of the two molecules in each complex are similar except for some residues of L-A and L-E in the 8-oxo-dGTP complex (root mean square deviation (r.m.s. deviation)^5^ is 0.62 Å for the corresponding 146 Cα atoms in the 8-oxo-dGTP complex, and 0.61 Å for the corresponding 146 Cα atoms in the 2-oxo-dATP complex). In the following text, we focus on one molecule in the asymmetric unit that has clearer electron densities of the ligands. hMTH1 adopts an α-β-α sandwich structure composed of two α-helices (α-1 and α-2) and eight β-strands (β-1 to β-8). The Nudix motif (23 residues from Gly-37 to Leu-59) is located at the center of the molecule and adopts a strand-loop-helix-loop structure that is formed by β-3′, L-B, α-1, and L-C. 8-Oxo-dGTP and 2-oxo-dATP bind to the same substrate binding pocket adjacent to the catalytic Nudix motif, which consists of β-1, β-3, β-3′, β-4, β-5, and α-2, and is surrounded by L-A (Fig. 1*A*). The overall structures of the 8-oxo-dGTP and 2-oxo-dATP complexes are similar except for the conformational difference of L-A and L-E with r.m.s. deviation of 0.34 Å for the corresponding 146 Cα atoms (Fig. 1*B*). In the 2-oxo-dATP complex, the electron densities of L-A are weak as compared with those in the 8-oxo-dGTP complex.

##### Recognition of 8-Oxo-dGTP and 2-Oxo-dATP

**FIGURE 2.**
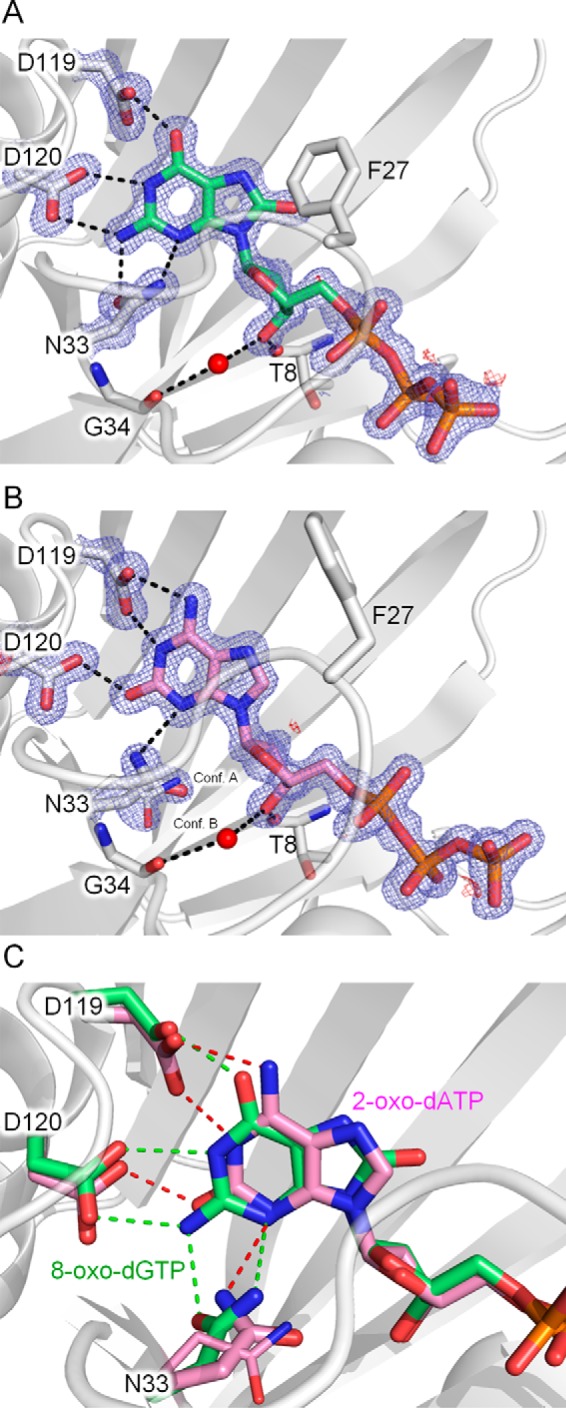
**Recognition of 8-oxo-dGTP and 2-oxo-dATP by hMTH1.** *A,* hydrogen-bonding interactions around 8-oxo-dGTP are shown as *dashed lines*. 8-Oxo-dGTP (*green*) and the amino acids (*white*) involved in the recognition are shown in *stick* representation. *B,* hydrogen-bonding interactions around 2-oxo-dATP. 2-Oxo-dATP is shown in *pink*. The 2*F_o_* − *F_c_* electron density maps (*blue*) are contoured at 1.2σ, whereas the *F_o_* − *F_c_* electron density maps (*red*) are contoured at 3.0σ. *C,* comparison of hydrogen bonding interactions between the 8-oxo-dGTP and 2-oxo-dATP complexes.

hMTH1 recognizes 8-oxo-dGTP and 2-oxo-dATP with the *anti*-conformation about the glycosidic bond in the substrate binding pocket. Despite the difference in pH, the recognition of the 8-oxoguanine (8-oxoG) base of 8-oxo-dGTP is quite similar to that observed in the previously reported structures of complexes with 8-oxo-dGMP or 8-oxo-dGTP (Fig. 2*A*) (29, 30): Asp-120 and Asn-33 form two hydrogen bonds (all distances 2.9 Å), and Asp-119 forms a single hydrogen bond (distance 2.5 Å) with 8-oxoG, respectively. In the three crystals, all distances of the corresponding hydrogen bonds are identical (differ by less than 0.1 Å). In the 2-oxo-dATP complex, on the other hand, 2-oxoadenine (2-oxoA) is recognized with a different hydrogen bonding pattern from that around 8-oxoG: the 2-oxoA base is recognized via two hydrogen bonds with Asp-119 and a single hydrogen bond with Asn-33 and Asp-120, respectively (Fig. 2*B*). The O2 atom of 2-oxoA, which is the characteristic feature of 2-oxoA, is recognized via a single hydrogen bond with Asp-120 (distance 2.6 Å). In addition, Asp-119 forms two hydrogen bonds with N1 and N6 (both distances 2.8 Å) of 2-oxoA. The side chain of Asn-33 shows two conformations, but the major conformation forms a single hydrogen bond (distance 3.0 Å) with N3 of the base. Accordingly, the position of 2-oxoA is shifted toward Asp-119 by the two hydrogen bonds with Asp-119 as compared with the position of 8-oxoG in the 8-oxo-dGTP complex where Asp-119 forms a single hydrogen bond with 8-oxoG (Fig. 2*C*). The geometries of key hydrogen bonds are summarized in supplemental Table S1.

These different recognition modes of hMTH1 for 8-oxoG and 2-oxoA seem to be consistent with the previously published mutational results showing that the substitution of alanine or asparagine for Asp-119 abrogates the 2-oxo-dATPase activity but preserves the 8-oxo-dGTPase activity (26). Trp-117 is also involved in the recognition of 2-oxoA with a π-π stacking interaction as in 8-oxoG. These results are consistent with the absence of 8-oxo-dGTPase and 2-oxo-dATPase activities in the W117A mutant, but it is not easy to explain why the W117Y mutant retains only 2-oxo-dATPase activity (26). There is a slight difference between the positions of 8-oxoG and 2-oxoA (Fig. 2*C*), which may indicate that some short contacts occur only during the recognition of 8-oxo-dGTP by the W117Y mutant. In the 8-oxo-dGTP complex, Phe-27 contributes to the affinity for 8-oxoG via the interaction with the O8 atom of 8-oxoG (Fig. 2*A*). On the other hand, Phe-27 in the 2-oxo-dATP complex is far away from the 2-oxoA base, and the electron densities of L-A including Phe-27 are less obvious than those in the 8-oxo-dGTP complex, *i.e.* L-A in the 2-oxo-dATP complex is more flexible. This result is consistent with the mutational analysis showing that the F27A mutation affects *K_m_* for 8-oxo-dGTP more than that for 2-oxo-dATP (26). hMTH1 shows 17-fold higher affinity (ΔΔ*G* = −1.7 kcal/mol) for 8-oxoG than for G (24) because of the favorable van der Waals interactions (through Met-81 and Phe-27) with the bulky O atom (8-oxoG) as compared with the H atom (G) at C8. The *anti-*glycosidic conformation observed in the crystals is preferred by G rather than by 8-oxoG nucleotides (ΔΔ*G* ≈ 1.4 kcal/mol) (8). Thus, the favorable van der Waals interactions can be estimated at ∼3 kcal/mol. In contrast, *E. coli* MutT exhibits high substrate specificity for 8-oxoG nucleotides. The structural basis has been reported by Nakamura *et al.* (8). MutT binds to 8-oxoG nucleotides with the large ligand-induced conformational change, and strictly recognizes the overall conformation of 8-oxoG nucleotides with the *syn-*glycosidic conformation through a number of hydrogen bonds. The hydrogen bonds between the pyrimidine moiety and the main chain atoms of Phe-35 discriminate the guanine base from the other normal and 2-oxoA bases and the hydrogen bonds between O6 and N7 of 8-oxoG and the amide group of Asn-119 do the 8-oxoG base from the guanine base.

**FIGURE 3.**
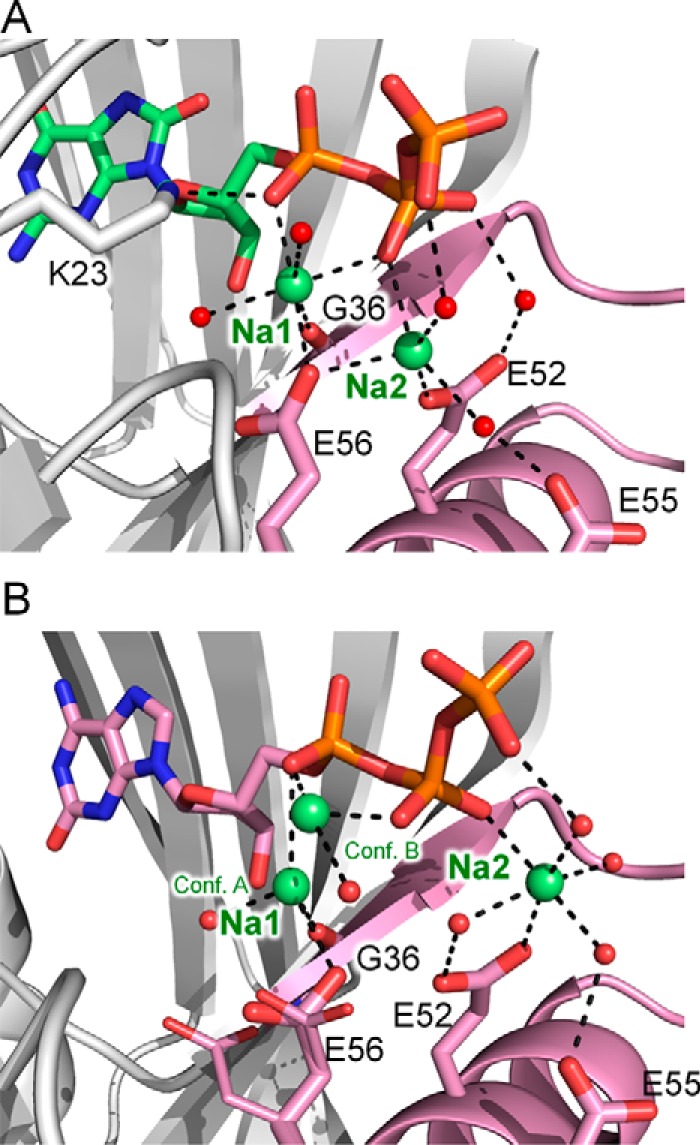
**Binding mode of the ligands' triphosphate moiety at the Nudix motif.** *A,* the 8-oxo-dGTP complex. *B,* the 2-oxo-dATP complex. The Nudix motif is shown in *pink*. Gly-36, Glu-52, Glu-55, Glu-56, and Lys-23 are shown as *stick* representations. Na^+^ ions and water molecules are shown as *green* and *red spheres*, respectively.

During the binding of the triphosphate moiety of 8-oxo-dGTP, Pα-O is recognized via a single hydrogen bond with the side chain of Lys-23. The β- and γ-phosphates of 8-oxo-dGTP bind to the Nudix motif through Na^+^ ions with unambiguous electron densities in our crystals at neutral pH (Figs. 2, *A* and *B*, and 3, *A* and *B*), whereas the 8-oxo-dGTP complex at pH 4.0 shows weak electron densities of the triphosphate moiety (unpublished structure) (31). One Na^+^ (Na1) forms coordinate bonds with Pα-O, Pβ-O, Gly-36, and Glu-56, whereas the other (Na2) forms coordinate bonds with Pβ-O, Glu-52, and Glu-56. Glu-55 forms a hydrogen bond with a water molecule coordinated with Na2. Gly-36, Glu-52, and Glu-56 were reported to be necessary for the 8-oxo-dGTPase activity (33, 34), indicating that the binding mode of β- and γ-phosphates reflects active conformations for the hydrolysis. We confirmed by crystallography that the hydrolysis of 8-oxo-dGTP in the crystal proceeds as a result of soaking of the 8-oxo-dGTP complex crystals in a MgCl_2_ solution.^6^ The weak electron densities of the triphosphate moiety observed at pH 4.0 may be the result of protonation of the Glu cluster in the Nudix motif. The binding mode of the triphosphate moiety of 2-oxo-dATP is basically similar to that of 8-oxo-dGTP despite the difference in the coordination of Na^+^ ions and the loss of interaction with Lys-23 (Fig. 3*B*).

##### Bond Length Analysis of Asp-119 and Asp-120 in the Binary Complexes of hMTH1

Our structural analysis revealed differences in the binding mode of 8-oxo-dGTP and 2-oxo-dATP by hMTH1 especially regarding to the hydrogen binding pattern of the two neighboring aspartic residues (Asp-119 and Asp-120). Accordingly, the elucidation of the protonation state of these two residues is important to improve our understanding of the hMTH1 mechanism. Although neutron crystallography is a reliable technique for locating H atoms and determination of the protonation states in protein, this technique requires relatively large crystals that are usually challengeable (35, 36). A bond length analysis is an alternative method to determine the protonation states of ionizable residues in proteins using high-resolution X-ray structures (37, 38). The method bases on the difference at a statistical significant level in bond length between the C—Oδ1 and C—Oδ2 bonds, where the C=O and C—OH bond lengths are expected to be 1.2 and 1.3 Å, respectively, in the carboxyl group, while the C—Oδ1 and C—Oδ2 bonds have similar bond lengths (1.25 Å) in the case of the delocalized carboxylate group. The accuracy of this method is highly dependent on the data resolution, completeness, and *B* factor of the target residue.

The 8-oxo-dGTP complex was refined at 1.21-Å resolution with 100% completeness and 2.8 *I*/σ*I* value in the outer shell, and the 2-oxo-dATP complex was at 1.20-Å resolution with low 81.9% completeness and 1.4 *I*/σ*I* value in the outer shell (Table 1). These final structures were further refined under unrestrained conditions by using SHELXL. As the results, in the case of the 2-oxo-dATP complex, the bond length analysis showed that both Asp residues were deprotonated. We could not judge whether the result was true or not, because our X-ray data with low 81.9% completeness and 1.4 *I*/σ*I* value in the outer shell were not satisfied with the criteria required for the bond length analysis method. Then, we crystallized again the 2-oxo-dATP complex using the hMTH1(G2K/C87A/C104S) mutant to improve the crystal quality and determined the structure at 1.00-Å resolution, which is referred to as the 2-oxo-dATP complex (high resolution). The structures of the 8-oxo-dGTP complex and the 2-oxo-dATP complex (high resolution) enable us to apply the bond length analysis for determination of the protonation state. Asp-119 and Asp-120 buried in the hMTH1 structure have low *B*-factors (average 14.1 Å^2^ in the 8-oxo-dGTP complex and average 6.4 Å^2^ in the 2-oxo-dATP complex (high resolution)). In the 8-oxo-dGTP complex, Asp-119 showed 1.31 (3) and 1.20 (3) Å for the C—Oδ1 and C—Oδ2, respectively. These results indicated that Asp-119 is protonated in the 8-oxo-dGTP complex. Although Asp-120 did not show a significant difference in the C—Oδ bond lengths (1.20 (3) and 1.25 (3) Å, respectively), indicating that Asp-120 is deprotonated.

**TABLE 1:** **Data collection and refinement statistics of hMTH1** Highest resolution shell is shown in parentheses.

	8-Oxo-dGTP complex	2-Oxo-dATP complex	2-Oxo-dATP complex*^a^* (high resolution)
**Data collection**			
Beamline	PF BL1A	PF BL1A	SPring-8 BL44XU
Wavelength (Å)	1.1	1.1	0.9
Space group	*P*2_1_2_1_2_1_	*P*2_1_2_1_2_1_	*P*2_1_2_1_2_1_
Unit cell parameters (Å)	*a* = 46.5, *b* = 47.6, *c* = 123.8	*a* = 46.5, *b* = 47.3, *c* = 123.7	*a* = 46.6, *b* = 47.3, *c* = 123.8
Resolution range (Å)	50.0–1.21 (1.23–1.21)	50.0–1.20 (1.22–1.20)	50.0–1.00 (1.02–1.00)
Observed reflections	521,662	511,717	1,386,904
Unique reflections	84,388	85,333	144,098
Completeness (%)	100 (100)	99.0 (81.9)	97.0 (94.2)
*R*_merge_ (%)*^b^*	5.3 (43.3)	5.2 (50.8)	6.3 (23.7)
〈*I*/σ〉	33.7 (2.8)	23.4 (1.4)	68.3 (11.1)

**Refinement statistics**			
Resolution range (Å)	50.0–1.21	50.0–1.20	50.0–1.00
No. of reflections used	84,297	85,229	144,022
Completeness (%)	100	99.5	97.0
*R*_work_/*R*_free_ (%)*^c^*	13.6/17.2	14.3/17.6	11.7/13.8
R.m.s. deviation in bonds (Å)	0.007	0.007	0.009
R.m.s. deviation in angles (deg.)	1.123	1.167	1.456
Ramachandran plot			
Favored (%)	98.2	99.4	98.5
Allowed (%)	1.8	0.6	1.5

*^a^* Using the triplet hMTH1(G2K/C87A/C104S) mutant.

*^b^ R*_merge_ = 100 Σ_*hkl*_ Σ*_i_* |*I_i_*(*hkl*) − 〈*I*(*hkl*)〉|/Σ_*hkl*_ Σ*_i_ I_i_* (*hkl*), where *I_i_*(*hkl*) is the observed intensity and 〈*I*(*hkl*)〉 is the mean value of *I_i_*(*hkl*).

*^c^ R*_work_ = 100 Σ‖*F_o_*| − |*F_c_*‖/Σ|*F_o_*|. *R*_free_ was calculated from the test set (5% of the total data).

The improvement in resolution of the 2-oxo-dATP complex (high resolution) compared with the 8-oxo-dGTP complex by 0.2 Å significantly affects on reducing the associated bond length standard deviations (from 0.03 to 0.01 Å), which results in high precision analysis. In this case, a residual peak around Oδ2 of Asp-120 is observed in the resulting *F_o_* − *F_c_* electron density map contoured at 3.0σ (supplemental Fig. S1), indicating its protonated state. Although the difference in the C—Oδ bond lengths of Asp-120 was 0.04 Å (1.23 (1) and 1.27 (1) Å for the C—Oδ1 and C—Oδ2 bond, respectively), Oδ2 seems to be protonated due to the smaller associated standard deviations as well as the peak in the *F_o_* − *F_c_* electron density map. On the other hand, Asp-119 did not show a significant difference in the C—Oδ bond lengths (1.26 (1) and 1.25 (1) Å). Accordingly, we proposed an alternative protonation state of the two aspartate residues (Asp-119 and Asp-120) in the binding pocket of hMTH1, and validated our proposed mechanism of hMTH1 by the kinetic evaluation of the Asp-120 mutants and the structural analysis of their complexes.

##### Kinetic Analysis of hMTH1 and the Asp-120 Mutants

**FIGURE 4.**
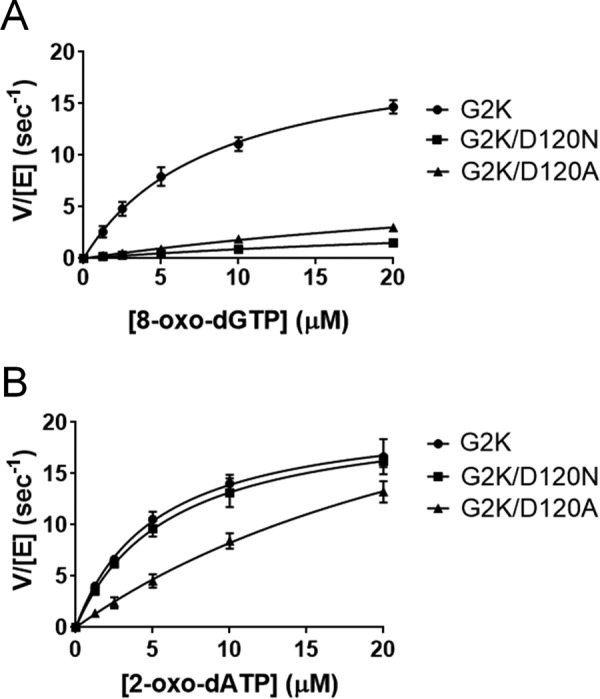
**Enzymatic activities of hMTH1 mutants.** *A,* the 8-oxo-dGTPase activities of the hMTH1 mutants. G2K, G2K/D120N, and G2K/D120A activities are presented as *circles*, *squares*, and *triangles*, respectively. *B,* the 2-oxo-dATPase activities of the hMTH1 mutants. Data from three independent experiments are presented as: *V* (hydrolyzed substrate (μm) per s) per [enzyme] (μm) *versus* [substrate] (μm). The Michaelis-Menten equation was applied to saturation curves using GraphPad Prism software.

To validate the catalytic contribution of Asp-120, which was identified for the first time as a residue important for the recognition of 8-oxo-dGTP and 2-oxo-dATP, we performed a kinetic analysis of two Asp-120 mutants: G2K/D120N and G2K/D120A (referred to as D120N and D120A, respectively). The initial velocities of 8-oxo-dGTP and 2-oxo-dATP hydrolysis by purified wild type, D120N, and D120A were determined in the presence of various concentrations of substrates (up to 20 μm) at pH 7.5 and 22 °C. The kinetic parameters were calculated using the Michaelis-Menten equation (Fig. 4, *A* and *B*) and are summarized in Table 2. The *k*_cat_/*K_m_* value, which represents the relative efficiency of the reaction, was found to be 2.5 and 4.1 μm^−1^ s^−1^ for 8-oxo-dGTPase and 2-oxo-dATPase of the wild type, respectively, confirming that hMTH1 hydrolyzes 2-oxo-dATP more efficiently than 8-oxo-dGTP, as reported elsewhere (23, 26). In terms of the 8-oxo-dGTPase activity, D120N and D120A showed only 4 and 8% of the activity of the wild type, respectively, because of their high *K_m_* and low *k*_cat_ values. These results suggest that the negatively charged carboxyl group of the side chain of Asp-120 is crucial for the recognition of 8-oxo-dGTP. On the other hand, for the 2-oxo-dATPase activity, the *k*_cat_/*K_m_* values of D120N and D120A are 3.5 and 1.1 μm^−1^ s^−1^: these levels correspond to 85 and 27% of the wild type activity, respectively. The substitution of Asp-120 with Ala decreases the affinity for 2-oxo-dATP 6-fold, but the D120N mutant retains the 2-oxo-dATPase activity similar to that of the wild type, with comparable *K_m_* and *k*_cat_, indicating that Asp-120 is protonated during the recognition of 2-oxo-dATP. The protonation state of Asp-120 and Asp-119 in the substrate binding pocket is discussed in detail later.

**TABLE 2:** **Kinetic parameters of the hMTH1 and the Asp-120 mutants** Reaction mixtures, containing various concentrations of substrates (0–20 μm for 8-oxo-dGTP or 2-oxo-dATP), were incubated with 1 nm hMTH1 or the Asp-120 mutants for 6 min at 22 °C. Reactions were terminated by heating to 99 °C for 10 min. *K_m_* and *k*_cat_ were obtained from Michaelis-Menten equation by GraphPad Prism software.

Mutation of hMTH1	Substrate	*K_m_*	*k*_cat_	*k*_cat_*/K_m_*
		μ*m*	*s*^−*1*^	μ*m*^−*1*^*s*^−*1*^
**Wild type (G2K)**	8-Oxo-dGTP	8.4 ± 0.9*^a^* (1.0)*^b^*	20.7 ± 1.0 (1.0)	2.5 (1.0)
	2-Oxo-dATP	5.2 ± 0.6 (1.0)	21.1 ± 0.9 (1.0)	4.1 (1.0)
**D120N mutant (G2K/D120N)**	8-Oxo-dGTP	41.2 ± 12.9 (4.9)	4.6 ± 1.1 (0.2)	0.1 (0.04)
	2-Oxo-dATP	6.0 ± 0.5 (1.2)	21.1 ± 0.8 (1.0)	3.5 (0.85)
**D120A mutant (G2K/D120A)**	8-Oxo-dGTP	44.4 ± 13.7 (5.3)	9.7 ± 2.2 (0.5)	0.2 (0.08)
	2-Oxo-dATP	31.8 ± 6.6 (6.1)	34.4 ± 4.9 (1.6)	1.1 (0.27)

*^a^* Each experiment was independently repeated. Reported results are mean ± S.D. from three independent experiments.

*^b^* Data in parentheses represent the relative values of each parameter in comparison to those of G2K.

##### The Relationship between Structure and Enzymatic Activity in the Asp-120 Mutants in Complex with 8-Oxo-dGTP or 2-Oxo-dATP

To validate the correlations between the structure and kinetics in the Asp-120 mutants, crystal structures of the Asp-120 mutants (D120N and D120A) in complex with 8-oxo-dGTP or 2-oxo-dATP were determined, respectively (Table 3). The results revealed that the Asp-120 mutation does not induce significant conformational changes except for L-A involved in the 8-oxoG recognition; the r.m.s. deviation value is 0.33–0.57 Å for the corresponding 146 Cα atoms in the 8-oxo-dGTP complexes and 0.15–0.17 Å for the corresponding 146 Cα atoms in the 2-oxo-dATP complexes.

**TABLE 3:** **Data collection and refinement statistics of the Asp-120 mutants** Highest resolution shell is shown in parentheses.

	D120N·8-oxo-dGTP complex	D120N·2-oxo-dATP complex	D120A·8-oxo-dGTP complex	D120A·2-oxo-dATP complex	D120A·2-oxo-dATP complex (high concentration)
**Data collection**					
Beamline	SPring-8 BL44XU	SPring-8 BL44XU	SPring-8 BL44XU	SPring-8 BL44XU	SPring-8 BL44XU
Wavelength (Å)	0.9	0.9	0.9	0.9	0.9
Space group	*P*2_1_2_1_2_1_	*P*2_1_2_1_2_1_	*P*2_1_2_1_2_1_	*P*2_1_2_1_2_1_	*P*2_1_2_1_2_1_
Unit cell *a*, *b*, *c* (Å)	45.8, 47.8, 123.6	46.2, 47.3, 123.2	46.3, 47.4, 123.1	46.2, 47.2, 123.2	46.4, 47.3, 123.4
Resolution range (Å)	50.0–1.50 (1.53–1.50)	50.0–1.39 (1.41–1.39)	50.0–1.19 (1.21–1.19)	50.0–1.19 (1.21–1.19)	50.0–1.18 (1.22–1.18)
Observed reflections	304,484	379,776	619,797	615,504	632,612
Unique reflections	44,359	53,100	87,153	86,636	89,484
Completeness (%)	99.7 (99.8)	96.3 (94.2)	99.5 (98.4)	99.9 (100)	99.6 (100)
*R*_merge_ (%)*^a^*	7.6 (29.1)	6.8 (29.9)	7.6 (35.5)	8.4 (62.7)	6.4 (34.4)
〈*I*/σ*I*〉	47.4 (9.3)	49.9 (7.7)	47.1 (6.4)	37.2 (3.0)	51.5 (8.3)

**Refinement statistics**					
Resolution range (Å)	50.0–1.50	50.0–1.39	50.0–1.19	50.0–1.19	50.0–1.18
No. of reflections used	44,286	53,052	87,075	86,548	89,390
Completeness (%)	99.5	96.3	99.5	99.7	99.5
*R*_work_/*R*_free_ (%)*^b^*	17.3/19.6	14.9/18.9	14.9/17.7	14.7/17.9	14.6/18.0
R.m.s. deviation bonds (Å)	0.006	0.006	0.008	0.007	0.007
R.m.s. deviation angles (deg.)	0.927	0.997	1.135	0.979	1.080
Ramachandran plot					
Favored (%)	99.7	99.1	99.7	99.1	99.4
Allowed (%)	0.3	0.9	0.3	0.9	0.6

*^a^ R*_merge_ = 100 Σ_*hkl*_ Σ*_i_*|*I_i_*(*hkl*) − 〈*I*(*hkl*)〉|/Σ_*hkl*_ Σ*_i_ I_i_*(*hkl*), where *I_i_*(*hkl*) is the observed intensity and 〈*I*(*hkl*)〉 is the mean value of *I_i_*(*hkl*).

*^b^ R*_work_ = 100 Σ‖*F_o_*| − |*F_c_*‖/Σ|*F_o_*|. *R*_free_ was calculated from the test set (5% of the total data).

**FIGURE 5.**
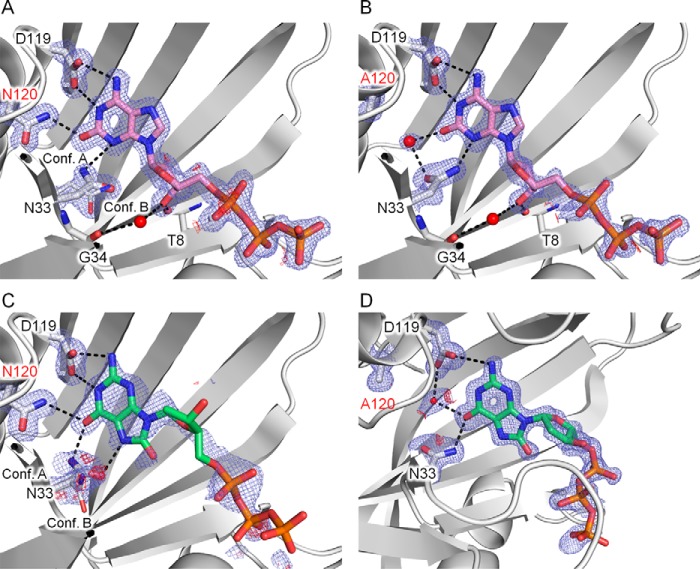
**Recognition of 2-oxo-dATP and 8-oxo-dGTP by mutants D120N and D120A.** *A*, the D120N·2-oxo-dATP complex. *B,* the D120A·2-oxo-dATP complex (high concentration). *C,* the D120N·8-oxo-dGTP complex. *D,* the D120A·8-oxo-dGTP complex. The 2*F_o_* − *F_c_* electron density maps (*blue*) are contoured at 1.2σ (*A* and *B*) or 0.8σ (*C* and *D*), whereas the *F_o_* − *F_c_* electron density maps (*red*) are contoured at 3.0σ.

The D120N mutant retains a 2-oxo-dATPase activity similar to that of the wild type, and actually, the structure of D120N in complex with 2-oxo-dATP shows that the 2-oxo-dATP binding mode of D120N is practically identical to that of the wild type (Figs. 2*B* and 5*A*). In the D120N complex, the electron densities of 2-oxo-dATP are clear (Fig. 5*A*). The orientation of the amide group of Asn-120 was determined by analyzing the temperature factor values of the O and N atoms after refinements. Thus, O2 of 2-oxo-dATP forms a hydrogen bond with Nδ-H of Asn-120. Accordingly, Asn-120 in the D120N mutant plays the same role in the interaction with 2-oxoA, as Asp-120 does in the wild type complex. On the other hand, in the structure of D120A complexed with 2-oxo-dATP, there is no electron density of 2-oxo-dATP, but there are electron densities of water molecules in the substrate-binding site (supplemental Fig. S2). Our kinetic analysis showed that D120A has 6-fold higher *K_m_* compared with the wild type (Table 2), and crystals of the D120A·2-oxo-dATP complex were prepared using a higher concentration of 2-oxo-dATP (see “Materials and Methods”). The structure of D120A·2-oxo-dATP (high concentration) shows a binding position of 2-oxo-dATP almost similar to that in the wild type and the electron densities of 2-oxo-dATP are clear (Fig. 5*B*). Instead of the disruption of the hydrogen bond between Asp-120 and 2-oxoA observed in the wild type complex, a water molecule (which is close to the substituted Ala-120) binds to 2-oxoA and Asn-33 (Fig. 5*B*). The loss of the direct interaction between 2-oxoA and Asp-120 explains the lower affinity of D120A for 2-oxo-dATP (Table 2). The similar and somewhat high *k*_cat_ values for 2-oxo-dATP in D120N and D120A, as compared with the wild type, are consistent with almost the same position of triphosphate in the three complexes.

**FIGURE 6.**
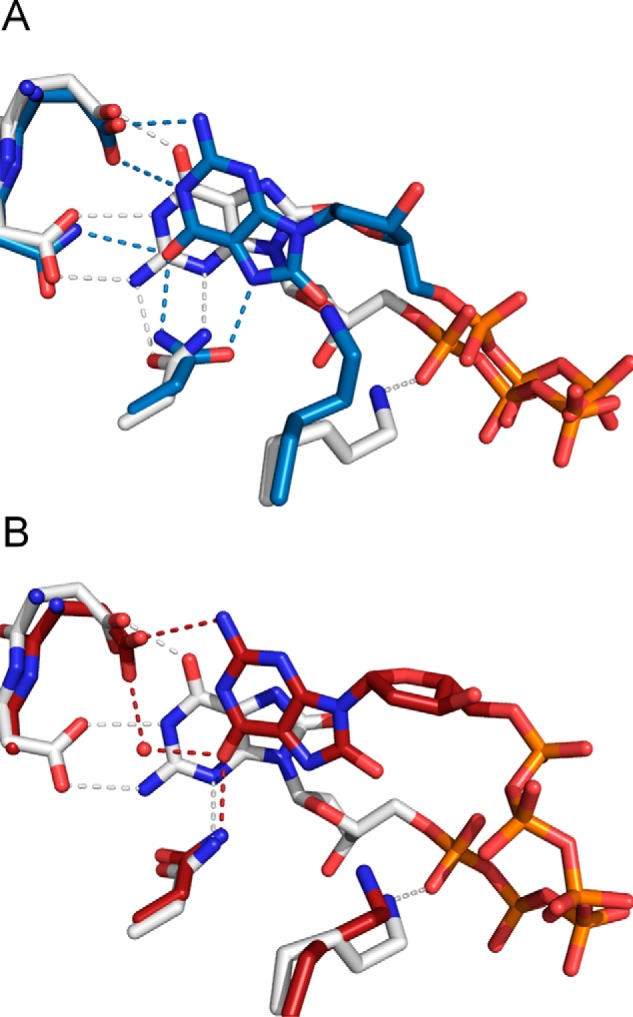
**Comparison of the 8-oxo-dGTP recognition between the wild type and mutants.** *A,* comparison between the wild type (*white*) and D120N (*blue*). *B*, comparison between the wild type (*white*) and D120A (*red*).

In contrast, during the recognition of 8-oxo-dGTP, the structures of both mutants (D120N and D120A) show modes of binding of 8-oxo-dGTP that are different from that of the wild type (Fig. 5, *C* and *D*). The face of 8-oxoG and the direction of deoxyribose are completely turned over in comparison with those in the wild type complex: this phenomenon is the result of the loss of two hydrogen bonds between Asp-120 and 8-oxoG (Fig. 6, *A* and *B*). In the D120N complex, Asp-119 and Asn-120 interact with H-N2-C2-N1-H and O6 of 8-oxoG, respectively, whereas Asn-33 with O6 and N7-H of 8-oxoG. In the case of D120A, because the hydrogen bond between Asn-120 and O6 of 8-oxoG observed in the D120N complex is lost, O6 of 8-oxoG is recognized via a hydrogen bond with Asn-33 and a water-mediated hydrogen bond with Asp-119 instead. Therefore, the 8-oxoG moiety shifts toward the solvent region in the D120A mutant compared with D120N. The 3′-OH group of deoxyribose in both complexes is directed away from Thr-8 and Gly-34, which are involved in the recognition of 3′-OH in the wild type. These findings are in agreement with the higher *K_m_* values of both mutants for 8-oxo-dGTP in comparison with the wild type. The electron densities of 8-oxo-dGTP in both mutants are ambiguous as compared with those in the wild type (Fig. 5, *C* and *D*). This result indicates lower occupancy and/or loose binding of 8-oxo-dGTP. The differences in the recognition of the 8-oxoG and deoxyribose moieties between the wild type and the mutants give rise to the difference in the position of the triphosphate moiety in 8-oxo-dGTP between the mutant structures and the original ones (active configuration bound to the Nudix motif) in the wild type structure (Fig. 6, *A* and *B*). The structural arrangement of the triphosphate reflects the lower *k*_cat_ values in both mutants. Nevertheless, it is surprising that the weak 8-oxo-dGTPase activity persists in the mutants (Table 2). This finding suggests that the triphosphate moiety can rarely move to the catalytically active position even in the case of the Asp-120 mutants.

##### The Protonation State of Asp-119 and Asp-120 and Insights into the Broad Substrate Specificity of hMTH1

**FIGURE 7.**
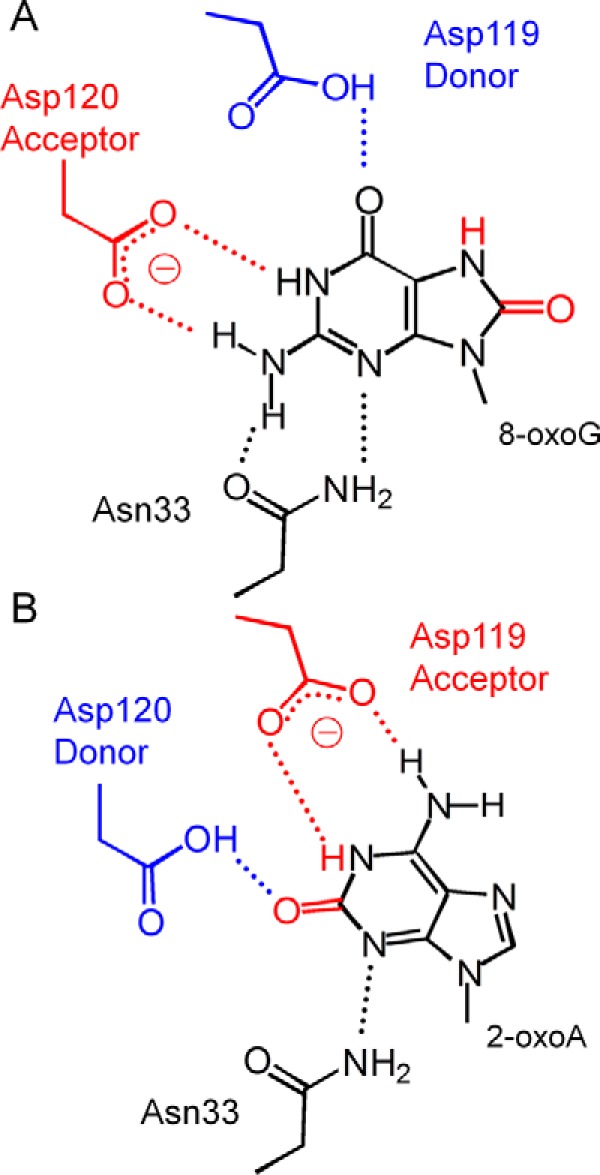
**Schematic views of the hydrogen donor and acceptor relationship between Asp-119 and -120 in recognition of 8-oxoG or 2-oxoA.** *A,* the 8-oxoG base in the keto form is recognized via protonated Asp-119 and deprotonated Asp-120. Asp-119 (*blue*) donates a hydrogen to O6 of 8-oxoG via its protonated carboxyl side chain, and Asp-120 (*red*) accepts two hydrogen atoms from N1 and N2 of 8-oxoG. Hydrogen bonds are shown as *dashed lines. B,* the 2-oxoA base in the keto form is recognized via deprotonated Asp-119 and protonated Asp-120. Asp-120 (*blue*) donates a hydrogen to O2 of 2-oxoA by its protonated carboxyl side chain, and Asp-119 (*red*) accepts two hydrogen atoms from N1 and N6 of 2-oxoA.

Our structural and kinetic data in this study and previously published mutational analysis enable us to understand the protonation state of neighboring aspartic residues (Asp-119 and Asp-120) involved in substrate recognition. Accordingly, we can also gain insights into the broad substrate specificity of hMTH1. During the recognition of 8-oxoG, Asp-120 forms two hydrogen bonds, and its substitution with the Asn residue, which acts as a hydrogen bond donor and acceptor, decreases the 8-oxo-dGTPase activity to 4%. This result supposes that Asp-120 differs from Asn, is deprotonated, and forms hydrogen bonds with N1-H and N2-H of the keto form of 8-oxoG (Fig. 7*A*) as expected from the p*K_a_* calculation for the guanine and 8-oxoguanine model compounds (30). Accordingly, Asp-119 is a hydrogen bond donor and recognizes O6 of 8-oxoG via the protonated -COOH side chain; this notion is validated by the finding that the D119N mutant retains 8-oxo-dGTPase activity (26). Therefore, we propose that hMTH1 recognizes the keto form of 8-oxoG via protonated Asp-119 and deprotonated Asp-120. In contrast, during the recognition of 2-oxoA, the protonation states of Asp-119 and Asp-120 are switched in comparison with the recognition of 8-oxoG. The D120N mutant shows a 2-oxo-dATPase activity comparable with that of the wild type, and the crystal structure of D120N in complex with 2-oxo-dATP shows that Nδ-H of Asn-120 forms a hydrogen bond with O2 of 2-oxoA (Fig. 5*A*). These results indicate that protonated Asp-120 acts as a hydrogen bond donor to interact with O2 of the keto form of 2-oxoA (Fig. 7*B*) (39). The hydrogen atoms at N1 and N6 of 2-oxoA participate in hydrogen bonds with deprotonated Asp-119 (Fig. 7*B*). This result is consistent with findings in other mutational studies showing that the D119N mutant completely loses the 2-oxo-dATPase activity (26), *i.e.* hMTH1 with the protonated form of Asp-119 may not bind to 2-oxo-dATP.

We emphasize that the exchange of the protonation state between Asp-119 and Asp-120 is required for the broad substrate specificity of hMTH1. Usually a carboxyl group is deprotonated at neutral pH. However, p*K_a_* values of carboxyl groups in proteins are affected by their environments such as solvent accessibility, electrostatic potential, and hydrogen bonds. The relationship between protein structure and ionization of carboxyl groups that was studied in 24 proteins indicates that almost all carboxyl groups with high p*K_a_* values (>5.5) are located in active sites or ligand-binding sites, and these carboxyl groups are buried and accept no more than one hydrogen bond (40). Asp-119 and Asp-120 of hMTH1 are also located and buried in the ligand-binding site, and they are involved in less than two intramolecular hydrogen bonds, supporting our structural finding that Asp-119 or Asp-120 are protonated at physiological pH and that the exchange of the protonation state contributes to the broad substrate specificity.

Here, we carried out structural and kinetic analyses of hMTH1 and demonstrated that hMTH1 shows broad substrate specificity for 8-oxo-dGTP and 2-oxo-dATP because of the exchange of the protonation state at Asp-119 and Asp-120 depending on the substrate. Recently, hMTH1 received attention as a new target for cancer treatments, and reported structures of hMTH1 in complex with various ligands also indicated that Asp-119 and Asp-120 are the key binding residues for design of small molecule inhibitors (16, 17, 22, 30, 41). These complex structures also suggest that the protonation site at Asp-119 and Asp-120 depends on the ligand (supplemental Fig. 3, *A–E*). The mechanism behind the broad substrate specificity of hMTH1 revealed in this study may facilitate the development of potent hMTH1 inhibitors.

## Materials and Methods

### 

#### 

##### Mutagenesis and Protein Expression and Purification

D120N or D120A mutations were introduced using the KOD-Plus-Mutagenesis Kit (Toyobo). The pET8c plasmid encoding hMTH1(G2K) served as a template (32). PCRs were performed with appropriate primers: 5′-AACAGCTACTGGTTTCCACTCCTGCT-3′ for the D120N mutant and 5′-CAGCTACTGGTTTCCACTCCTGC-3′ for the D120A mutant (the mutation sites are underlined), with 5′-ATCGGGCCACATGTCCTTGAAGGG-3′ as a general reverse complementary primer for both mutants. The PCR products were digested with DpnI to eliminate the template plasmid. The mutated sites were verified by sequencing. The hMTH1(G2K/C87A/C104S) mutant gene was synthesized by consideration of *E. coli* codon usage and cloned into the NdeI and XhoI sites of pET24a. The expression and purifications of hMTH1(G2K), hMTH1(G2K/D120N), hMTH1(G2K/D120A), and hMTH1(G2K/C87A/C104S) were conducted as described elsewhere (32). The purified proteins were concentrated to 10 mg/ml in a buffer consisting of 20 mm HCl, pH 7.5, 1 mm EDTA, 5% glycerol, and 1 mm β-mercaptoethanol.

##### Crystallization, Data Collection, and Structure Refinement

Crystallization was performed as described elsewhere (32). 8-Oxo-dGTP and 2-oxo-dATP were purchased from TriLink BioTechnologies and Jena Bioscience, respectively. All the crystals except D120A · 2-oxo-dATP (high concentration) were grown in a droplet that contained 0.5 μl of 10 mg/ml of protein, 0.5 μl of 10 mm 8-oxo-dGTP or 2-oxo-dATP, and 1 μl of the reservoir solution (1.0 m sodium citrate, 0.1 m Tris-HCl, pH 7.0, and 0.2 m NaCl; or 1.0 m sodium citrate, 0.1 m cacodylate-HCl, pH 6.5, and 0.2 m NaCl) equilibrated against 0.5 ml of the reservoir solution. For crystallization of D120A·2-oxo-dATP (high concentration), 60 mm 2-oxo-dATP was used.

All the crystals were transferred into a cryoprotectant solution supplemented with 20% glycerol or 30% sucrose and flash frozen in a nitrogen stream at 100 K. X-ray diffraction experiments were carried out on beamlines BL1A at Photon factory (Tsukuba) and BL44XU at SPring-8 (Harima). The diffraction data were indexed, integrated, and scaled with HKL2000 (42), and converted to the mtz file format with CCP4 (43). The structures were refined using the PHENIX software (44) with our refined coordinates of the hMTH1·8-oxo-dGTP complex as a starting model (32) and the model building was carried out in COOT (45). Either TLS or anisotropic refinements were used depending on the resolution. All molecular graphics were prepared using the PyMOL software (46). The data collection and refinement statistics are listed in Tables 1 and 3. For the bond length analysis of aspartate residues, we carried out unrestrained refinements of the final structures for the three binary complexes (Table 1) using SHELXL (47) to get reliable distances and estimate the associated standard deviations (37, 38).

##### Measurement of 8-Oxo-dGTPase and 2-Oxo-dATPase Activities

Catalytic activities of hMTH1 and its mutants were assayed in reaction mixtures (volume 100 μl) containing 0.1 m Tris acetate, pH 7.5, 40 mm NaCl, 10 mm magnesium acetate, 2 mm dithiothreitol, 0.005% Tween 20, and up to 20 μm nucleotide substrate (8-oxo-dGTP or 2-oxo-dATP). Each purified protein was added (1 nm), and the reaction mixtures were incubated at 22 °C for 6 min. After that, the reactions were terminated by heating at 99 °C for 10 min. Pyrophosphate (PP_i_) produced by the activity of hMTH1 or its mutants was detected using the PP_i_ Light Inorganic Pyrophosphatase Assay Kit (Lonza Rockland Inc.). The bioluminescent signal was measured using a Tecan Infinite M1000, and the amount of PP_i_ produced was calculated using a PP_i_ standard curve. The *K_m_* and *k*_cat_ values and their standard errors were estimated using nonlinear regression of the initial rate (s^−1^) of the reactions *versus* the concentration of the substrate using the Michaelis-Menten equation in GraphPad Prism software.

## Author Contributions

S. W., T. N., Y. N., and Y. Y. designed the research project. S. W., T. N., K. H., Y. K., M. C., T. A., T. T., and S. I. performed experiments. S. W., T. N., and Y. Y. wrote the manuscript. All authors analyzed the results, and approved the final version of the manuscript.

## Supplementary Material

Supplemental Data

## Footnotes

6 T. Nakamura, K. Hirata, S. Waz, M. Chirifu, S. Ikemizu, and Y. Yamagata, unpublished results.

## Footnotes

5 The abbreviations used are:

r.m.s. deviation: root mean square deviation
hMTH1: human MutT homolog 1
8-oxoG: 8-oxoguanine
2-oxoA: 2-oxoadenine
Nudix: nucleoside diphosphate linked to some other moiety, X.
