# Supplementary material for: Structural and Kinetic Studies of the Human Nudix Hydrolase MTH1 Reveal the Mechanism for Its Broad Substrate Specificity
Source: J Biol Chem. 2016 Dec 29;292(7):2785–94. doi: 10.1074/jbc.M116.749713 (PMC5314174; doi:10.1074/jbc.M116.749713)
Supplement: Supplemental Data [file supp_292_7_2785__index.html]

Structural and Kinetic Studies of the Human Nudix Hydrolase MTH1 Reveal the Mechanism for Its Broad Substrate Specificity — Broad Substrate Specificity of hMTH1 — Supplemental Data 

# Structural and Kinetic Studies of the Human Nudix Hydrolase MTH1 Reveal the Mechanism for Its Broad Substrate Specificity

## Supplemental Data

- Supplemental Data
