# Supplementary material for: Structural and Kinetic Studies of the Human Nudix Hydrolase MTH1 Reveal the Mechanism for Its Broad Substrate Specificity
Source: J Biol Chem. 2016 Dec 29;292(7):2785–94. doi: 10.1074/jbc.M116.749713 (PMC5314174; doi:10.1074/jbc.M116.749713)
Supplement: Supplemental Data [file supp_M116.749713_Supp_Figures.pdf]

Structural and Kinetic Studies of the Human Nudix Hydrolase MTH1 Reveal the Mechanism for Its Broad Substrate Specificity

Shaimaa Waz[1a], Teruya Nakamura[1,2a], Keisuke Hirata[1], Yukari Koga-Ogawa[1b], Mami Chirifu[1], Takao Arimori[3c], Taro Tamada[3], Shinji Ikemizu[1], Yusaku Nakabeppu[4] and Yuriko Yamagata[1*]

Supplementary Fig. 1. The electron density maps in the structure of the 2-oxo-dATP complex (high resolution). The $2F_o$-F_c electron density map is shown in blue (1.2σ). The F_o-F_c electron density map is shown in red (3.0σ, marked with a red arrow).

Supplementary Fig. 2. Electron densities of the D120A–2-oxo-dATP complex (low conc.). The $2F_o$-F_c electron density map (blue) is contoured at 1.2σ and the F_o-F_c electron density map (red) is contoured at 3.0σ.

Supplementary Fig. 3. Proposed protonation sites in the reported hMTH1-ligand complexes. (A) The hMTH1–8-oxo-dGTP complex. (B) The hMTH1–2-oxo-dATP complex. (C) The hMTH1–(S)-crizotinib complex (1). (D) The hMTH1–TH288 complex (2). (E) The hMTH1–(S)-4 complex (3).

References

1. Huber, K. V. M., Salah, E., Radic, B., Gridling, M., Elkins, J. M., Stukalov, A., Jemth, A.-S., Göktürk, C., Sanjiv, K., Strömberg, K., Pham, T., Berglund, U. W., Colinge, J., Bennett, K. L., Loizou, J. I., Helleday, T., Knapp, S., and Superti-Furga, G. (2014) Stereospecific targeting of MTH1 by (S)-crizotinib as an anticancer strategy. *Nature* **508**, 222-227
2. Gad, H., Koolmeister, T., Jemth, A.-S., Eshtad, S., Jacques, S. A., Ström, C. E., Svensson, L. M., Schultz, N., Lundbäck, T., Einarsdottir, B. O., Saleh, A., Göktürk, C., Baranczewski, P., Svensson, R., Berntsson, R. P. A., Gustafsson, R., Strömberg, K., Sanjiv, K., Jacques-Cordonnier, M.-C., Desroses, M., Gustavsson, A.-L., Olofsson, R., Johansson, F., Homan, E. J., Loseva, O., Bräutigam, L., Johansson, L., Höglund, A., Hagenkort, A., Pham, T., Altun, M., Gaugaz, F. Z., Vikingsson, S., Evers, B., Henriksson, M., Vallin, K. S. A., Wallner, O. A., Hammarström, L. G. J., Wiita, E., Almlöf, I., Kalderén, C., Axelsson, H., Djureinovic, T., Puigvert, J. C., Häggblad, M., Jeppsson, F., Martens, U., Lundin, C., Lundgren, B., Granelli, I., Jensen, A. J., Artursson, P., Nilsson, J. A., Stenmark, P., Scobie, M., Berglund, U. W., and Helleday, T. (2014) MTH1 inhibition eradicates cancer by preventing sanitation of the dNTP pool. *Nature* **508**, 215-221
3. Streib, M., Kräling, K., Richter, K., Xie, X., Steuber, H., Meggers, E. (2013) An organometallic inhibitor for the human repair enzyme 7,8-dihydro-8-oxoguanosine triphosphatase. *Angew Chem. Int. Ed. Engl.* **53**, 305-309

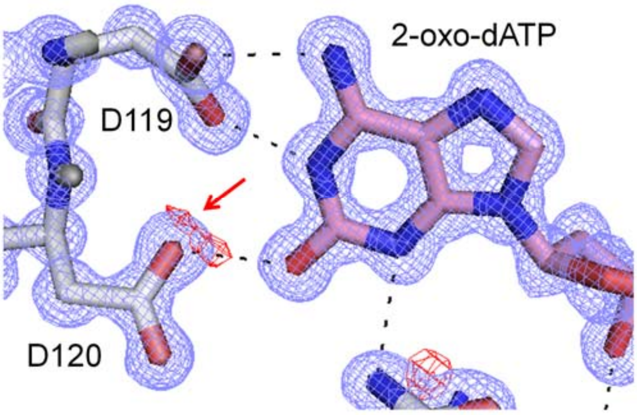

Supplementary Fig.1

D120A-2-oxo-dATP, Mol A

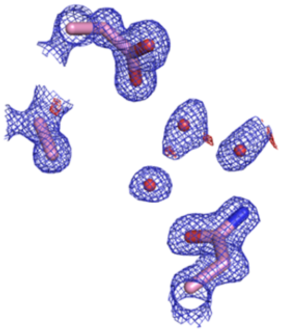

Supplementary Fig.2

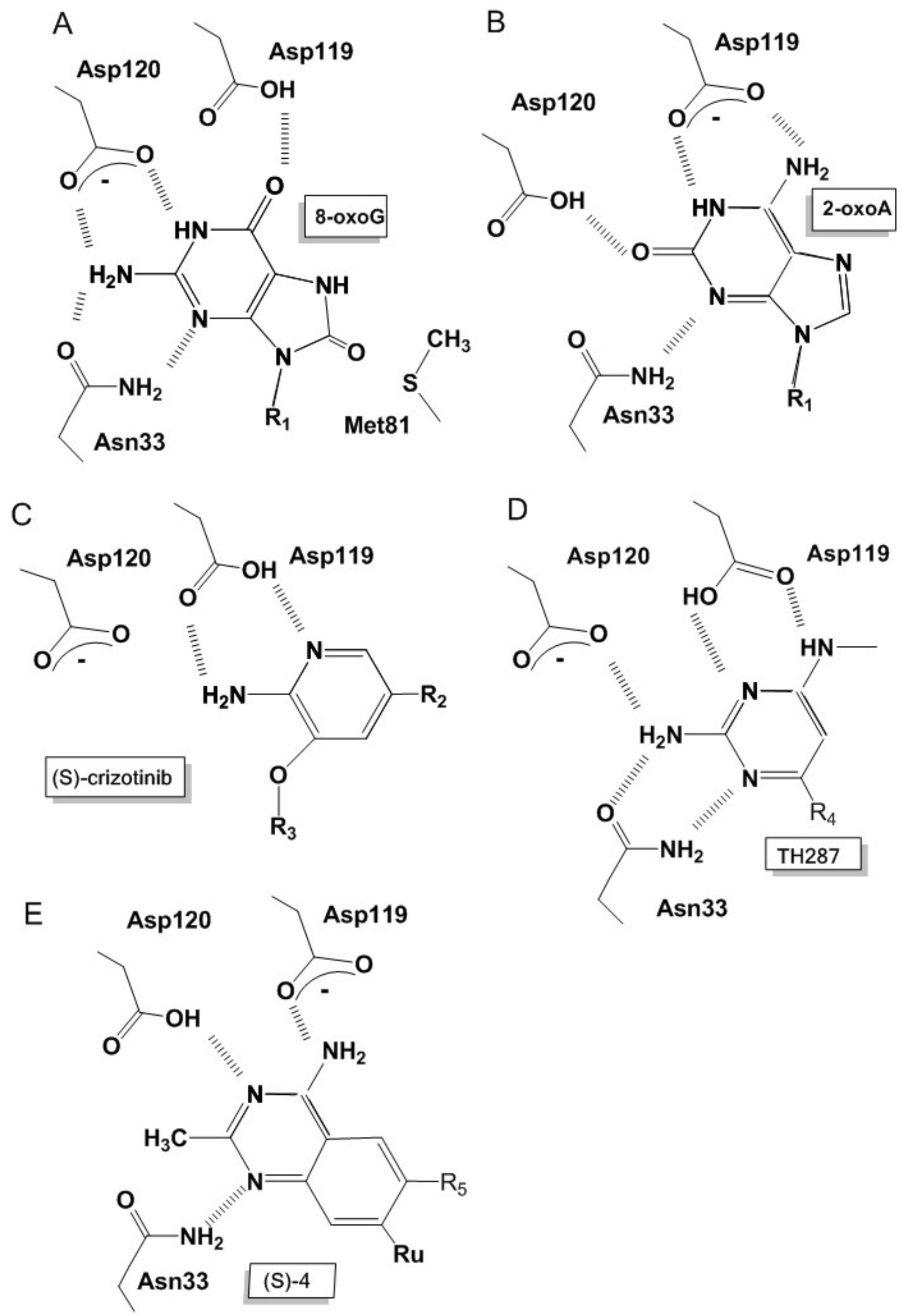

Supplementary Fig.3

Supplementary Table (1)
Geometry of hydrogen bonds.

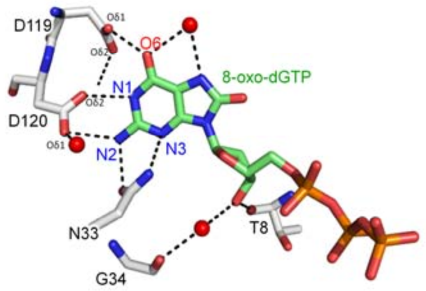

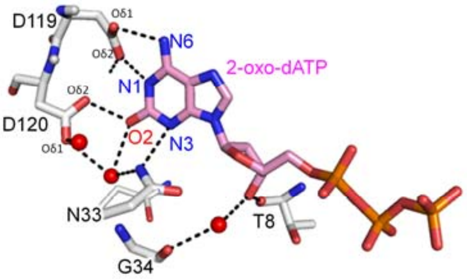

Structure	Residue	Hydrogen bonds	Distance[#] (Å)	Angle(θ1°)*	Angle(θ2°)**
**8-oxo-dGTP complex**	Asp119	-COOH-----O6 of 8-oxoG	2.5	134	112
	Asp120	-COδ2----N1H of 8-oxoG	2.9	132	139, 100
	Asp120	-COδ1----$N2H_2$ of 8-oxoG	2.9	98	129
	Asn33	-COδ-----$N2H_2$ of 8-oxoG	2.9	111	112
	Asn33	-CNδ-----N3 of 8-oxoG	2.9	113	112
	Thr8	-CO-----3'OH of ribose	2.7		
	Gly34	-CO----Wat----3'O of ribose	2.8, 2.9		
**2-oxo-dATP complex**	Asp119	-COδ1-----$N6H_2$ of 2-oxoA	2.8	112	107
	Asp119	-COδ2-----N1H of 2-oxoA	2.8	109	118
	Asp120	-COOH-----O2 of 2-oxoA	2.6	124	118
	Asn33	-CNδ-----N3 of 2-oxoA	2.9	96, 125	119
	Thr8	-CO-----3'OH of ribose	2.7		
	Gly34	-CO----Wat----3'O of ribose	2.9, 2.8		
**D120N-8-oxo-dGTP complex**	Asp119	-COδ1-----$N2H_2$ of 8-oxoG	2.7	125	100
	Asp119	-COδ2-----N1H of 8-oxoG	3.2	91	118
	Asn120	-CNδ-----O6 of 8-oxoG	3.3	120	125
	Asn33	-COδ-----N7 of 8-oxoG	2.7	114	115, 130
	Asn33	-CNδ-----O6 of 8-oxoG	2.9	125	120
**D120N-2-oxo-dATP complex**	Asp119	-COδ1----$N6H_2$ of 2-oxoA	2.8	116	110
	Asp119	-COδ2----N1H of 2-oxoA	2.9	109	117
	Asn120	-CNδ----O2 of 2-oxoA	2.8	123	124
	Asn33	-CNδ----N3 of 2-oxoA	3.0	90,126	113
	Thr8	-CO----3'OH of ribose	2.7		
	Gly34	-CO----Wat----3'O of ribose	2.9, 2.8		
**D120A-8-oxo-dGTP complex**	Asp119	-COδ1----$N2H_2$ of 8-oxoG	2.9	138	103
	Asp119	-COδ2---Wat---O6 of 8-oxoG	2.8, 2.7		
	Asn33	-CNδ-----O6 of 8-oxoG	2.8	124	125
**D120A-2-oxo-dATP complex**	Asp119	-COδ1-----$N6H_2$ of 2-oxoA	2.9	112	106
	Asp119	-COδ2-----N1H of 2-oxoA	2.7	110	114, 121
	Asn33	-COδ1----Wat----O2 of 2-oxoA	2.8, 2.5		
	Asn33	-CNδ-----N3 of 2-oxoA	2.8	108, 106	132
	Thr8	-CO-----3'OH of ribose	2.7		
	Gly34	-CO----Wat----3'O of ribose	2.8, 2.9		

[#]Distance between two heteroatoms
* θ1 angle between AA-A--D and ** θ2 angle between DD-D—A. Where A is an acceptor atom, D is a donner atom, AA is an acceptor antecedent, and DD is a donner antecedent.
